# From Across the Seas

**Published:** 1916-04-15

**Authors:** 


### FROM ACROSS THE SEAS.

### Meat Prices; Cooking; The Staff's Liabilities.

As the first newspaper published in the world dealing mainly with hospitals, their evolution, progress, administration, and needs, we have long wished to give our readers some idea of the developments in hospital literature which have taken place in recent years. We hope this object may be met by the new section, of which we publish the first article to-day. Our contemporaries necessarily have a special interest for the Editor of The Hospital, which, we hope, may be extended, in some measure at any rate, through this new department, which it will be our endeavour to make as interesting and practical as possible.

### Meat Prices in Australia.

The hospital caterer will learn with interest from the *Prince Alfred Hospital Gazette*, of Sydney, that meat-buying is no less difficult in a great meat-producing dominion, such as Australia, than it is at home. While a hospital contract for bread in Melbourne has been made for 1916 on the basis of 5⅝d. for a 4-lb. loaf, the following are the prices paid per lb. for meat as compared with the two previous years:—

| | 1914 | 1915 | 1916 |
|---|---|---|---|
| Beef— | | | |
| Rump steak ... ... ... ... | 6d. | 6d. | 9d. |
| Steak ... ... ... ... ... | 4½d. | 4½d. | 6½d. |
| Corned round ... ... ... | 4½d. | 5d. | 7d. |
| Mutton— | | | |
| Chops ... ... ... ... ... | — | 5½d. | 6d. |
| Sides ... ... ... ... ... | 3¼d. | 3¾d. | 5½d. |
| Legs ... ... ... ... ... | 3¾d. | 4½d. | 6½d. |
| Lamb— | | | |
| Sides ... ... ... ... ... | 4d. | 4½d. | 6d. |
| Joints ... ... ... ... ... | — | — | 5½d. |
| Pork ... ... ... ... ... | 7d. | 10d. | 9d. |

Bought wholesale, by the body (or portion of a body), prices in Sydney have also ruled high this year, as will be learned from the figures here quoted:

| | 1914 | 1915 | 1916 |
|---|---|---|---|
| Beef, Quarters ... ... ... | 1d. | 3½d. | 5½d. |
| Beef, Ribs and Loins ... ... | 4¼d. | 6½d. | 10½d. |
| Sheep ... ... ... ... ... | 3d. | 3½d. | 5¼d. |

It will be seen that in two years quarters of beef have risen nearly 500 per cent., ribs and loins nearly 150 per cent., and mutton 70 per cent.

### "Crux Medicorum."

An interesting case touching upon the responsibility of the honorary medical staff of a hospital as regards accidents to patients is reported in the *Medical Journal of South Africa*, Johannesburg. The facts are briefly as follows: A woman was admitted into the Pretoria Hospital as an ordinary patient under the honorary surgeon on duty. An operation was performed after which, while in bed and still under the influence of the anæsthetic, the woman sustained a burn from a hot-water bag applied to her leg by a nurse acting under the authority of the ward sister. After the patient's discharge an action for damages was brought against the hospital committee on the ground of culpable negligence on the part of the doctors and nurses in attendance at the operation—the impression of the patient apparently being that the injury was sustained on the operation-table.

Judgment was delivered by Mr. Justice Wessels, concurred in by Mr. Justice Mason and Mr. Justice Curtewis, and the main points were as follows:—

(1) The hospital committee is only a committee of management and administration. The honorary medical officers are not the employers of the hospital committee, and over them the hospital committee has no control. Therefore the relation of principal and agent does not exist between the hospital committee and the doctors and surgeons who perform their duties at the hospital.

(2) Though the resident medical officers are appointed by the committee, and receive their emoluments through the committee, yet this committee is not responsible for their professional acts.

(3) Similarly, if a nurse is engaged at an operation under the supervision of the medical officer who is actually in charge of the operation, she is not, during this time, the employee or agent of the hospital committee, and the hospital committee is not liable for her acts, although at other times the hospital committee may be liable.

Whatever may be the legal aspect of the situation created by this judgment, it seems inequitable that the honorary staff of a hospital, who are doing their work gratuitously, should be made liable for the heavy expenses which are likely to accrue from undertaking the defence of actions of this nature. The action in question cost the Pretoria Hospital Committee, notwithstanding that they won the case, some £230. How can medical staffs be best protected? Some form of insurance would possibly be the best solution, and it seems likely that hospital committees will be asked to provide this or some other form of indemnification.

### Institutional Cooking.

We all know the wonderful culinary achievements possible by the agency of a frying-pan or of a gipsy fire, but it is to be hoped that the results of the other extreme of equipment, that of the up-to-date institutional kitchen, are not to be condemned on the opinion of a writer in the *Hospital World*, of Toronto, who unburdens his mind thus:

Hospital kitchens are equipped with spotless and up-to-date utensils and modern conveniences—porcelain soup-kettles, marble kneading-tables, nickel dishes and covers, marvellous food-chests and refrigerators—a place of white and gold and silver shining. And yet out of it comes coarse soup, flavourless meats, heavy vegetables, and sloppy puddings, not for the delectation of the hospital staff alone—that would not matter so much, but primarily for the nourishment of enfeebled bodies, and to make appeal to the capricious invalid appetite that turns from all but the most delicate dishes.

Institutional cooking! We all know what the term implies. The condition is bad enough in the county jail and the poor-house; but in a modern hospital it is or ought to be an impossible state of affairs.

FROM ACROSS THE SEAS (continued).

### A Warning to Casualty Officers.

Too many institutions are finicky about the kind of cases they take. Hospitals are refuges to which sick people may go in the hope that they will be taken in and that there will be proper treatment to get them well again. Sick people do not choose the disease from which they suffer, and generally when they approach a hospital they do not know what is the matter with them. If, when they arrive at the institution and are looked over by a young medical man, it is found that they have any one of half a dozen so-called infectious or contagious diseases, they are refused admission.

This is all wrong, and the kind of hospital that picks and chooses its cases ought to be out of business; either its policy is too narrow and its administrators and supporters are too selfish, or it has not the means to permit it to give a proper hospital service to the community, and it ought to be closed up. The hospital that cannot accept a sick person because of any particular disease has no right to exist in this modern day; any hospital is liable to be overcrowded and almost any hospital can be crowded in some one or another department, but it is inconceivable that any acute disease hospital should turn away any acute disease for any other reason.—*The "Modern Hospital," St. Louis.*

